# Mr. Little, on Strangulated Hernia

**Published:** 1807-01

**Authors:** D. Little

**Affiliations:** Surgeon. Plymouth Dock


### *To the Editors of the Medical and Physical Journal.*

Gentlemen,

In the course of my practice, it has fallen to my lot to meet with a very considerable number of Cases of Strangulated Hernia, in the treatment of which, I have some little reason to believe, I have been very successful, in reducing them by the usual means.—If the treatment of the following Case has any thing in it which may be deemed worthy of a place in your valuable and widely extended Journal, by an employment of a powerful agent in a way that I am not aware of its having been, and your inserting it, will oblige Your most obedient servant,

D. LITTLE, Surgeon.

*Plymouth Dock, Oct.* 27, 1806.

---

On the morning of Oct. 23, 1806, John Coxe, a post boy, aged about 30, subject to severe Asthma, applied to me with a Strangulated Inguinal Hernia, which had come down about three hours before. The complaint had existed rather more than three years, from a blow by a horse, but had never been troublesome, being easily reducible by gentle pressure and an horizontal posture.

The pain was excessive; from the moment of strangulation, the stomach immediately became disordered, and threw up its contents; a stool had been passed a short time previous to its descent.

I attempted reduction, but was foiled in every endeavour. An opiate of gts. 50. of tinct. opii, with gts. 45 of tinct. Digitalis was then given, and a cold saturnine lotion applied to the part; this somewhat moderated the pain.—After an interval of six hours, I renewed my endeavours to reduce it, but in vain. I then threw up an injection of the infusion of tobacco, in the proportion of a drachm to a pint of water; in about ten or twelve minutes it began to nauseate; vomiting quickly ensued, with some languor; this moment was seized to endeavour to effect the reduction, but the stricture was so great, that it baffled all the address and skill which considerable experience in those cases had given me. Under these unpromising circumstances, the patient was made acquainted with the danger of his case; that it was more than probable, that nothing short of an operation was left for his relief.—I visited him again in about six hours, with my friend Mr. Dunning; at this moment it occurred to me, to use the affusion of cold water to the part, which was immediately done, by pouring a tea kettle full in a continued stream, from about the height of seven or eight feet over the scrotum and spermatic process. This occupied about eight or ten minutes. Its effects were evident on the general system, by reducing the pulse, as well as on the part itself, by lessening the Hernia, which, by gentle pressure, was soon and easily reduced, an immediate relaxation taking place on the strictured part.

I have every reason to ascribe the relief of my patient to the cold affusion, and as it has been seldom thus employed, I am induced to give it you.—I have only to add, that I hope future trial may confirm its utility; should it prove true, I shall feel highly gratified, in having suggested any means likely to relieve this formidable and dangerous accident.

